# Supplementary material for: Engineering Adenoviral Vectors with Improved GBM Selectivity
Source: Viruses. 2023 Apr 28;15(5):1086. doi: 10.3390/v15051086 (PMC10224093; doi:10.3390/v15051086)
Supplement: Supplementary file 1 [file viruses-15-01086-s001.zip › viruses-2346965-supplementary.pdf]

Table S1. Promoter sequences inserted into Ad5 vector expressing luciferase.

Promoter	5′-3′ Promoter Sequence
Survivin Promoter	CACGCGTTCTTTGAAAGCAG-TCGAGGGGGCGCTAGGTGTGGGCAGGGAC-GAGCTGGCGCGGCGTCGCTGGGTGCACCGCGACCACGGGCAGAGCCACGCGGCGGGAGGAC-TACAACTCCCGGCACACCCCGCGCCGCCCCGCCTC-TACTCCCAGAAGGCCGCGGGGGGTGGACCGCCTAAGAG-GGCGTGCGCTCCCGACATGCCCCGCGGCGCGCCATTAACCGCCAGATTTGAATCGCGGACCCGTTGG-CAGAGGTGGCGGCGGCGGCA
hTERT Promoter	CTTCCCACGTGGCGGAGGGACTGGGGACCCGGG-CACCCGTCCTGCCCCTTCAC-CTTCCAGCTCCGCCTCCTCCGCGCGGACCCCGCCCCGTCCCGACCCCTCCCGGGTCCCCGGCCCAGCCCCCTCCGGGCCCTCCCAGCCCCTCCCCTTCCTTTCCGCGGCCCCGCCCTCTCCTCGCGGCGCGAG-TTTCAGGCAGCGCTGCGTCCTGCTGCGCACGTGGGAA-GCCCTGGCCCCGGCCACCCCCGCG
Survivin/hTERT Promoter (Survivin+LUC+BHG polyA + hTERT)<br>BHG polyA	TGTGCCTTCTAGTTGCCAGCCATCTGTTGTTT-GCCCCTCCCCCGTGCCTTCCTTGACCCTG-GAAGGTGCCACTCCCACTGTCCTTTCCTAATAAAATGAGGAAATTGCATCGCATTGTCTGAGTAGGTGTCATTC-TATTCTGGGGGGTGGGGTGGGGCAG-GACAGCAAGGGGGAGGATTGGGAAGACAATAGCAGGCATGCTGGGGATGCGGTGGGCTCTATGGC
